# Nature-Inspired Optimization Algorithms for Neuro-Fuzzy Models in Real-World Control and Robotics Applications

**DOI:** 10.1155/2019/9128451

**Published:** 2019-04-15

**Authors:** Fevrier Valdez, Oscar Castillo, Amita Jain, Dipak K. Jana

**Affiliations:** ^1^Tijuana Institute of Technology, Tijuana, BC, Mexico; ^2^Ambedkar Institute of Advanced Communication Technologies and Research, Delhi, India; ^3^Haldia Institute of Technology, Haldia, India

Nature-inspired optimization algorithms are a recent topic of research, and they are based on using some nature-inspired behaviors to solve optimization problems. Currently, a large number of approaches have been developed in this area, such as particle swarm optimization, bat algorithm, ant colony optimization, bee colony, dolphin algorithm, wolf search, flower pollination algorithm, and cat swarm. However, how to design efficient nature-inspired algorithms and how to use these algorithms for real-world application problems in control and robotics are still important issues. In particular, the design of neuro-fuzzy models, like type 2 fuzzy neural networks, type 1 fuzzy neural models, and intuitionistic fuzzy neural networks, has some current interest. In addition, new emerging neural models have been recently proposed. In all these models, a common problem is how to obtain an optimal structure, which can be handled by nature-inspired optimization algorithms. This special issue aims to bring researchers to report their latest research work on development of new nature-inspired algorithms or innovative applications of existing algorithms in the design of neural models for real-world applications in control and robotics, with an ultimate goal of exploring future research directions. In this special issue, we have five papers selected after a careful reviewing process. The five papers are representative of the current state of the art in this area.

G. López-Vázquez et al. present a grammatical evolution- (GE-) based methodology to automatically design third-generation artificial neural networks (ANNs), also known as spiking neural networks (SNNs), for solving supervised classification problems. The proposal performs the SNN design by exploring the search space of three-layered feedforward topologies with configured synaptic connections (weights and delays) so that no explicit training is carried out. Besides, the designed SNNs have partial connections between input and hidden layers which may contribute to avoid redundancies and reduce the dimensionality of input feature vectors. The proposal was tested on several well-known benchmark datasets from the UCI repository and statistically compared against a similar design methodology for second-generation ANNs and an adapted version of that methodology for SNNs; also, the results of the two methodologies and the proposed one were improved by changing the fitness function in the design process. The proposed methodology shows competitive and consistent results, and the statistical tests support the conclusion that the designs produced by the proposal perform better than those produced by other methodologies.

On the other hand, R. Soto et al. presented a binary cat swarm optimization for solving the manufacturing cell design problem (MCDP). This problem divides an industrial production plant into a certain number of cells. Each cell contains machines with similar types of processes or part families. The goal is to identify a cell organization in such a way that the transportation of the different parts between cells is minimized. The organization of these cells is performed through cat swarm optimization, which is a recent swarm metaheuristic technique based on the behavior of cats. In that technique, cats have two modes of behavior: seeking mode and tracing mode, selected from a mixture ratio. For experimental purposes, a version of the autonomous search algorithm was developed with dynamic mixture ratios. The experimental results for both normal binary cat swarm optimization (BCSO) and autonomous search BCSO reach all global optimums, both for a set of 90 instances with known optima and for a set of 35 new instances with 13 known optima.

C. Zhang et al. proposed the ionic liquid gel (ILG), a new type of soft actuator material, which is a mixture of 1-butyl-3-methylimidazolium tetrafluoroborate (BMIMBF4), hydroxyethyl methacrylate (HEMA), diethoxyacetophenone (DEAP), and ZrO_2_ polymerized into a gel state under ultraviolet (UV) light irradiation. The soft actuator structure consists of a layer of ionic liquid polymer gel sandwiched between two layers of activated carbon capped with gold foil. The volume of the cationic BMIM+ in the ionic liquid BMIMBF4 is much larger than that of the anionic BF_4_^−^. When voltages are applied to both sides of the actuator, the anions and cations move toward the anode and cathode of the electrode, respectively, under the electric field. The volume of the ILG cathode side therefore expands and the volume of the ILG anode side shrinks, hence bending the entire actuator toward the anode side. The Ogden model was selected as the hyperelastic constitutive model to study the mechanical properties of the ILG by nonlinear analysis. As the ILG is an ideal material for the preparation of a supercapacitor, the equivalent circuit of the ILG can be modeled by the supercapacitor theory to identify the transfer function of the soft actuator. The central pattern generator (CPG) control is widely used in the area of biology, and CPGs based on bioinspired control methods have attracted great attention from researchers worldwide. After the continuum soft actuator is discretized, the CPG-based bioinspired method can be used to control the soft robot drivers. According to the simulation analysis results, the soft actuator can be smooth enough to reach the specified location.

Q. Zhu et al. proposed a novel motion planning method for autonomous ground mobile robot to address dynamic surroundings, nonlinear program, and robust optimization problems. A planner based on recurrent fuzzy neural network (RFNN) is designed to program trajectory and motion of mobile robot to reach target. And, obstacle avoidance is achieved. In RFNN, inference capability of fuzzy logic and learning capability of neural network are combined to improve nonlinear programming performance. Recurrent frame with self-feedback loops in RFNN enhances stability and robustness of the structure. Extended Kalman filter (EKF) is designed to train weights of RFNN considering kinematic constraint of autonomous mobile robot as well as target and obstacle constraints. EKF's characteristics of fast convergence and little limit in training data make it suitable to train the weights in real time. Convergence of the training process is also analyzed in this paper. Optimization technique and update strategy are designed to improve robust optimization of the system in dynamic surroundings. Simulation experiment and hardware experiment are implemented to prove effectiveness of the proposed method. Hardware experiment is carried out on a tracked mobile robot. Omnidirectional vision is used to locate the robot in the surroundings. Forecast improvement of the proposed method is then discussed at the end.

Finally, C. Sepúlveda et al. proposed a population pharmacokinetic (PopPK) model allowing the researchers to predict and analyze the drug behavior in a population of individuals and to quantify the different sources of variability among these individuals. In the development of PopPK models, the most frequently used method is the nonlinear mixed effect model (NLME). However, once the PopPK model has been developed, it is necessary to determine if the selected model is the best one of the developed models during the population pharmacokinetic study, and this sometimes becomes a multiple criteria decision making (MCDM) problem; frequently, researchers use statistical evaluation criteria to choose the final PopPK model. The use of the evaluation criteria mentioned above entails big problems since the selection of the best model becomes susceptible to the human error mainly by misinterpretation of the results. To solve the previous problems, we introduce the development of a software robot that can automate the task of selecting the best PopPK model considering the knowledge of human expertise. The software robot is a fuzzy expert system that provides a method to systematically perform evaluations on a set of candidate PopPK models of commonly used statistical criteria. The presented results strengthen our hypothesis that the software robot can be successfully used to evaluate PopPK models, ensuring the selection of the best PopPK model.

